# Dark matter RNA illuminates the puzzle of genome-wide association studies

**DOI:** 10.1186/1741-7015-12-97

**Published:** 2014-06-12

**Authors:** Georges St. Laurent, Yuri Vyatkin, Philipp Kapranov

**Affiliations:** 1 St. Laurent Institute, 317 New Boston St, Suite 201, Woburn, MA 01801, USA; 2 AcademGene Ltd., 6, Acad. Lavrentyev ave., Novosibirsk 630090, Russia

**Keywords:** Genome-wide association study, Non-coding RNA, vlincRNA, Intronic RNA, lncRNA, RNA scaffold, LincRNA, Long Non-coding RNA, Long intergenic non-coding RNA, Very long intergenic non-coding RNA

## Abstract

In the past decade, numerous studies have made connections between sequence variants in human genomes and predisposition to complex diseases. However, most of these variants lie outside of the charted regions of the human genome whose function we understand; that is, the sequences that encode proteins. Consequently, the general concept of a mechanism that translates these variants into predisposition to diseases has been lacking, potentially calling into question the validity of these studies. Here we make a connection between the growing class of apparently functional RNAs that do not encode proteins and whose function we do not yet understand (the so-called ‘dark matter’ RNAs) and the disease-associated variants. We review advances made in a different genomic mapping effort – unbiased profiling of all RNA transcribed from the human genome – and provide arguments that the disease-associated variants exert their effects via perturbation of regulatory properties of non-coding RNAs existing in mammalian cells.

## Introduction

**Figure 1:**
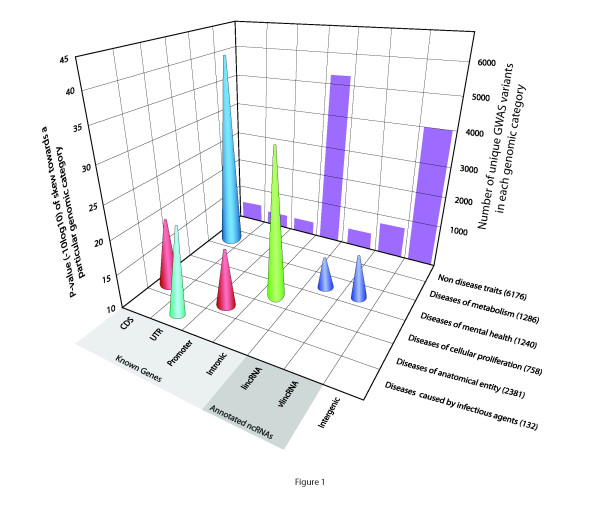
**Discovery of genome-wide association studies (GWAS) variants in different genomic elements and disease types.** GWAS variants were assigned to a disease type (*y* axis) or non-disease traits. For each disease type, the *P*-value (*x* axis) for a skew towards a particular genomic category (*x* xis) was calculated (see Additional file 1: Supplementary Text). Numbers of unique GWAS variants for each of the genomic categories are shown as the purple bars; the corresponding numbers for each of the disease types are shown in parenthesis. Only disease types with >100 GWAS variants are shown. CDS, coding DNA sequence (coding regions of known genes); UTR, untranslated region (non-coding regions of known genes; promoter and intronic regions are those of known genes). See Additional file 1: Supplementary Text for details of the analysis.

Connecting variations in DNA sequence with a biological or medical phenotype has long served to map functional elements of a genome. The recent genomics revolution has facilitated the identification of such variants on a massive scale, ushering in the era of genome-wide association studies (GWAS). Since the first pioneering report in 2005 [1], hundreds of such analyses have identified thousands of changes in DNA sequence (primarily single nucleotide polymorphisms (SNPs)) associated with a large number of complex diseases (cancers, heart disease, brain disorders, obesity, and many others; [2,3]. However, most of these variants have accumulated in unannotated, non-coding regions of the genome, whose functions continue to pose an enigma (Figure 1). Therefore, much of the wealth of GWAS information remains unrealized, with the mechanisms of action of the underlying genomic regions unknown, despite their widespread associations with disease.

### Pervasive transcription: the answer to function behind the non-coding GWAS variants?

Only 2 to 3% of human DNA (genome) encodes proteins, the building blocks of life whose function we understand fairly well. The remaining 97 to 98% represent non-coding sequences, which were long considered ‘junk DNA’ because they did not fit the protein-centric view that dominated biology for decades. The goal of connecting DNA sequence variants to this protein-coding sliver of the human genome has shaped their interpretation, yet over 90% of GWAS hits lie in the non-coding parts of the genome (Figure 1; see Additional file 2: Supplementary Tables 1–3). (Table 1). Three possible explanations exist for the large preponderance of non-coding GWAS hits. They could arise from methodological errors such as imprecise measurements of phenotypes [4], differences in population structures [5] or DNA quality issues [6,7] between cases and controls. Second, they could affect distal regulatory regions of known (mostly protein-coding) genes. Third, they could represent novel genes or transcripts. However, as the number of GWAS increase, support for the first two arguments continues to weaken. The sheer number of non-coding GWAS hits, their continued accumulation as statistical power has improved, and their consistent discovery across different diseases and different studies (Figure 1), argues against a widespread pattern of errors. Although errors must exist, they are unlikely to represent such a large proportion of GWAS events. Similarly, distal enhancers and regulatory regions will eventually explain some fraction of non-coding GWAS hits. However, these variants must interrupt fairly small sites of transcription factor binding and chromatin signaling within the regulatory regions, which represent a small minority of the genome. For example, Khurana *et al*. [8] reported that conserved transcription factor binding motifs and DNAse I hypersensitive regions make up only 0.4% of the genome. As expected, only 88 out of approximately 12,000 variants in the National Human Genome Research Institute GWAS catalog currently map to these regions. Therefore, the third explanation, that non-coding SNPs affect novel genes or transcripts, has begun to take center stage. In effect, the broad and continued accumulation of GWAS data, with the same pattern of distribution in non-coding regions, highlights the importance of pervasive transcription and dark matter RNA.

**Table 1:** Glossary of technical terms

Term	Meaning
Chromatin signaling	A system of regulation of gene activity in a cell that works by affecting the immediate surroundings of DNA, for example, by modifying various proteins that coat DNA inside the nucleus. Depending on the exact nature of the modification, DNA becomes either more or less accessible to cellular machinery that activates genes
Enhancer	A sequence of DNA that can regulate a target gene or genes over long distances
DNAse I hypersensitivity region	A region of DNA identified in an assay where chromatin is digested with DNAse I, an enzyme that degrades DNA. More accessible regions of chromatin, typically containing regulatory elements such as promoters and enhancers, are more susceptible to DNAse digestion and thus are enriched in DNAse I hypersensitivity regions
Gene Ontology (GO) term	GO is a an international initiative aimed at assigning controlled vocabulary, consisting of *terms* such as ‘regulation of apoptosis’ that define the functional property of each gene. This vocabulary is often very useful in understanding the biological meaning of a genomics experiment. For example, a list of genes activated during a disease would have a list of specific terms associated with each gene. Enrichment of specific terms in the list would suggest general cellular functions in which these genes participatem and give clues to the molecular functions underlying the disease
H1 embryonic stem cells	A line of human embryonic stem cells maintained in culture
H3K27 trimethylation	A certain type of chemical modification of a protein that binds DNA. Important for reversible deactivation oftargeted portions of the genome
Intron	Part of an RNA molecule that is included immediately after transcription and removed during maturation of that molecule
Intronic RNA	RNA encoded by a DNA sequence that also encodes an intron of another transcript
lincRNA-p21	A non-coding RNA activated upon DNA damage and in various tumor cell lines
*MYC* gene	A gene encoding an important regulator controlling activity of many genes. This gene has been associated with many cancers
Normal human epidermal keratinocytes (NHEK)	A line of primary keratinocytes maintained in culture
Non-coding RNA	RNA that is not used as a template for protein synthesis
Pervasive transcription	Massive transcription from unannotated regions of the genome
PolyA+ RNA	A molecule of RNA containing a long stretch of adenosine residues at the end
PRC2 chromatin signaling complex	A complex composed of multiple protein molecules that reversibly modifies chromatin and silences target genes
Promoter	A sequence of DNA that is located immediately adjacent to a target gene and regulates its activity
Pseudogene	A copy of a gene, presumed to be non-functional, although a number of recent examples describe both non-coding functions and occasionally coding functions for some of these loci
Regulation in *trans*	Regulation via interaction with molecules encoded by distal regions of the genome
RNA Pol II	A complex composed of multiple protein molecules responsible for synthesis of RNA, which is used as template for protein synthesis
Transcript	A molecule of RNA produced by transcription, that is, copying of RNA from the DNA template
Transcription factor	A protein that regulates expression of genes by binding to their promoters and/or enhancers
Transcription factor motif	A short DNA sequence recognized by a transcription factor or group of transcription factors, typically found in promoters and enhancers
Transcriptome	A collection of all the RNA molecules (transcripts) in a cell or a tissue
Transcriptomics	Study of the transcriptome
*Xenopus* oocytes	Oocytes from frogs of genus *Xenopus*, an important model system for study of developmental biology, cell biology, molecular biology, toxicology, and neuroscience

In 2002, the first report of pervasive transcription [9] subsequently triggered a series of genome-mapping endeavors that have discovered large numbers of dark matter RNAs transcribed from much of the non-coding space of the human genome [10-12]. Although an object of considerable debate over the last decade [13], an increasing number of independent observations in different species [14-19] have confirmed these results by continually increasing the annotation of the dark matter transcriptome. Presently, little doubt remains that the human genome produces large amounts of RNA whose function we still do not understand [10-12]. In fact, non-coding (nc)RNA represents at least 75% of the human genome [20], and its relative mass outweighs that of protein-coding mRNA [21].

These large and well-validated datasets now provide a strong basis for an in-depth look at ncRNA as a possible answer to the mystery of the many GWAS hits that do not fall neatly into protein-coding regions of the human genome. The non-coding disease-associated polymorphisms from the GWAS studies may have uncovered a vast hidden regulatory layer composed of ncRNA transcripts and their network of interactions in the cell. Below we describe the evidence supporting this view, and explain how this perspective can solve a number of outstanding questions.

### Undiscovered transcripts may underlie non-coding GWAS variants

As discussed by Mudge *et al*. [12] in their excellent review on functional transcriptomics, we have only begun to annotate the full complexity of RNAs encoded by the human genome. First, the database of complete, full-length cDNAs – the basis for gene annotations – still has surprisingly shallow coverage [10]. In fact, for many gene loci, the database contains only a single complete cDNA. This implies that most protein-coding genes, even well-characterized ones, have yet undiscovered exons that would require highly sensitive in-depth profiling methods to reveal [22,23]. With much less coverage than coding genes, the annotation situation for ncRNAs remains far more incomplete. The main reasons for this include the over-reliance on methods designed for protein-coding mRNAs, such as the use of polyA+ RNA for transcriptome profiling, the analytical focus on spliced RNAs, and the avoidance of intronic RNAs. The vast majority of RNA sequencing (RNAseq) experiments so far have profiled the polyA+ RNA fraction. Although this is informative for mRNAs, it leads to loss of significant complexity of ncRNAs [21], and a bias against the discovery of their unspliced versions. As a consequence, spliced versions of ncRNAs dominate the current annotated lists, which has resulted in an underestimate of their genomic coverage. It is very possible that, unlike protein-coding mRNAs, the longer, unspliced versions of the ncRNAs represent the functional forms. The abundance of ncRNAs in the nucleus compared to the cytosol [18] makes this a likely scenario.

Partly to bring order to this complexity, annotation efforts have classified ncRNAs by their physical characteristics. Typically, they are defined based on length (short, long, or very long), location relative to known genomic features (introns, genes, promoters, enhancers, or intergenic space), and overlap of known genes (sense or antisense) [12]. Of greatest importance to GWAS,long ncRNAs (lncRNAs) include all the classes of ncRNAs greater than 200 nucleotides in length, such as long intergenic ncRNAs (lincRNAs) [24], very long intergenic ncRNAs (vlincRNAs; (>40 kb in length, see below), natural antisense RNAs, and intronic RNAs [19,25-27]. Genomic annotation efforts typically focus on intergenic regions as the logical place to look for novel genes and transcripts, following the general notion that introns of known genes probably represent mere pre-mRNAs. However, this simple assumption has failed in the light of recent RNAseq datasets, as we have shown in a mouse inflammation time-course experiment [28]. In fact, thousands of mouse introns can harbor functional ncRNAs that behave separately from their exonic counterparts [28], resonating with discoveries of independent intronic RNAs in other systems such as *Xenopus* oocytes [29]. These observations support visionary ideas originally conceived by John Mattick almost 2 decades ago [30].

Strikingly, discovery of GWAS variants seem to favor different genomic elements depending on disease type (Figure 1). Only two disease types showed a preference for discovery of GWAS variants in coding regions of known genes (CDSs): metabolic diseases and anatomical diseases. By contrast, diseases affecting mental health favored annotated lncRNAs (lincRNAs and vlincRNAs), while cellular proliferation diseases favored introns (Figure 1). The even distribution in the non-disease trait category (which includes a large number of different phenotypes) provides a perspective for the contrasting results in the disease categories. Although understanding these observations will require additional research, we hope they raise the question of why variants in different diseases favor different categories of genomic elements. For example, it is tempting to speculate that the preference for promoters in anatomical diseases relates to the importance of tight control of gene expression during development.

As alluded to above, most of the existing lists of lncRNAs come from profiling of polyA+ RNA. By contrast, sequencing of total RNA from normal and tumor tissues recently uncovered vlincRNAs), a novel class of lncRNAs that showed statistically significant associations with GWAS variants [31]. Thousands of these vlincsRNAs span at least 10% of the human genome, and probably span much more, once additional tissues are profiled. These RNAs range from 40 kb up to around 1 MB in length, and are controlled by typical RNA Pol II promoters. Interestingly, an intriguing subset of these RNAs – those controlled by promoters within endogenous retroviral elements – characterizes cancerous and pluripotent states.

**Figure 2:**
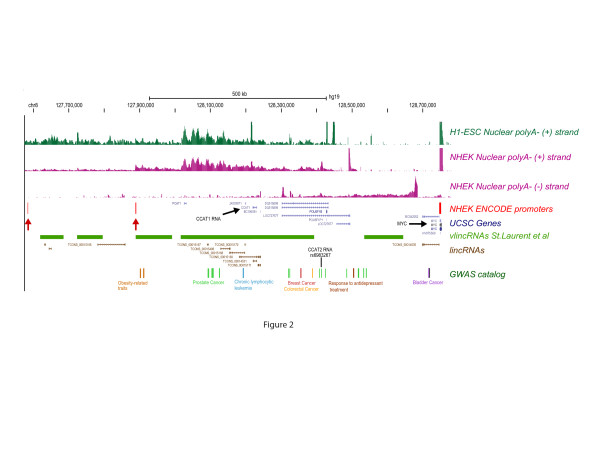
**A genomic view of the 8q24 region upstream of the *MYC *** **gene.** For details, see text.

Figure 2 illustrates a cancer-associated region, for which GWAS has highlighted the potential importance of vlincRNAs. This 8q24 region upstream of the *MYC* gene shows a high level of transcription in two normal human cell lines: H1 embryonic stem cells and normal human epidermal keratinocytes (NHEK), which are primary keratinocytes [20]. vlincRNA transcription occurs on both strands, probably in part from normal RNA Pol II promoters [32] (Figure 2, red arrows). In total, vlincRNAs [31] span 727 kb of that 1.2 MB region and overlap a number of GWAS SNPs. Lower level transcription overlaps additional GWAS SNPs, suggesting the presence of unannotated transcripts in those regions, consistent with the data in Figure 2. The two previously studied cancer-associated ncRNAs in this region, the 2,613 bp-long CCAT1 [33] and the 340 bp-long CCAT2 [34], represent only a tiny fraction of its transcriptional complexity. Quite possibly, they encompass only segments of much longer transcripts. In fact, the current profile of transcription leaves us with an attractive possibility that a cluster of GWAS SNPs spanning around 500 kb works via its presence in a small set of very long ncRNAs. Obviously, all this shows that we have only begun the exploration of RNAs made in this important region, and by association, many regions throughout the genome, where unexplained non-coding GWAS hits occur. Widespread presence of such very long RNAs suggests that vlincRNAs represent a global property of the human genome, and advances the theory that such RNAs mediate the functions of variants uncovered by GWAS (also see below).

### Emerging patterns of lncRNA function in disease

A number of recent examples indicate that ncRNAs can underlie the function of non-coding GWAS SNPs. Perhaps the best-studied example is ANRIL, a lncRNA transcribed from the 9p21.3 locus. the single greatest GWAS risk factor for atherosclerosis that is currently known [35]. Remarkably, the expression level of this ncRNA stands out as the variable most strongly associated with the disease phenotypes linked to 9p21.3 [35]. Recent work has highlighted the importance of the atherogenic SNPs in this locus by demonstrating that ANRIL functions by *trans-*regulating over 900 genes [36]. The study combined various *in vitro* experiments with measurement of gene expression in 2,280 patients with cardiovascular disease from the Leipzig Heart Study to show that these ANRIL-regulated genes can be classified into known atherogenic Gene Ontology terms such as ‘cell adhesion’ and ‘apoptosis’ [36].

The study further showed that ANRIL interacts with the PRC2 chromatin signaling complex, and requires an intact Alu sequence (a short repeated sequence present in thousands of copies in the human genome) for its regulatory effects [36]. Delivery of the PRC2 complex, which promotes H3K27 trimethylation and repression of gene expression, to its targets via the Alu-mediated interaction thus provides an attractive model for ANRIL function. Other systems have previously provided similar examples of Alu repeats mediating intermolecular interactions between RNA molecules, and leading to functional consequences [37,38]. All this evidence suggests that many other lncRNAs might function through intermolecular interactions mediated by Alu and other abundant repeated sequences in mammalian genomes.

The molecular mechanism of ANRIL function illustrates a potential general paradigm in gene expression regulation that promises to explain the function of large numbers of lncRNAs. In this model, one type of RNA species could regulate hundreds of targets in *trans* via intermolecular interactions, a mode of regulation previously associated primarily with short RNAs such as micro RNAs. However, it is becoming increasingly clear that lncRNAs can function in this manner, perhaps by providing scaffolds that bring together various protein and RNA molecules [39]. In this regard, the functions of ANRIL parallel those of the lincRNA HOTAIR, which also *trans-*regulates hundreds of genes by changing the chromatin occupancy of PRC2 [40].

A newly discovered vlincRNA associated with Hemolysis, elevated liver enzymes, low platelet count syndrome provides another prominent example of this mode of function [41]. While this syndrome represents a mendelian disorder, mapped using a traditional genetic analysis of affected families, the mutations occur in a non-coding region that harbors an ncRNA approximately 200 kb long. Subsequent analysis has shown that mutations can affect stability of this RNA [41], which also functions by *trans-*regulating hundreds of target genes.

Impressive as the aforementioned examples are, even more striking is the trend that has emerged from these and other investigations: more detailed examination of non-coding GWAS loci have increasingly led to the discovery of disease-relevant transcripts in the highlighted region. For example, a careful examination of the GWAS region at 5p14.1 that is implicated in autism led to the discovery of non-coding RNA antisense to a moesin pseudogene. Further experiments determined that this natural antisense RNA probably works in *trans* by lowering the level of moesin protein encoded by a gene on the X chromosome [42]. Supporting this emerging trend, depletion (small interfering RNA or antisense RNA) or over-expression of lncRNAs, even the ultra-long vlincRNAs, now routinely results in apparent phenotypes [31,41]. The growing wealth of examples precludes us from going into details of the studies or even citing all of them. Suffice to say that such functional analysis has begun to connect ncNAs, including those transcribed from GWAS loci, with processes such as cancer, heart disease, degenerative diseases, and senescence [31,36,40,41,43-45].

### A complex transcriptome for complex diseases

The magnitude of functional transcripts in non-coding genomic space, many of which still remain either hidden or under-appreciated as functional RNAs, makes it almost certain that these RNAs will explain an important fraction of the non-coding GWAS hits. If so, then further intriguing questions arise. Why are mendelian disorders mostly explained by mutations affecting protein-coding exons, yet complex diseases are explained by mutations in ncRNAs? Does this notion form a pattern consistent with prevailingmechanistic models of how ncRNAs function? Does this pattern tell us something about the underlying systems biology of human cells?

The evidence thus far suggests positive answers to these questions. As described above in the few examples that have been worked out in detail, lncRNAs can act as *trans* regulators, potentially master regulators, of large numbers of known genes. Examples such as ANRIL, HOTAIR, lincRNA-p21, and HELLP highlight this emerging paradigm. A similar model of regulation occurs with transcription factors; however, a mutation in a long ncRNA would generally not have quite the same effect as a mutation in a protein. Considering that the former usually results in a much more flexible phenotype than the latter, a mutation would affect rather than abrogate the interaction affinity of the lncRNA with its partner molecules, such as proteins or other nucleic acids. Moreover, these interactions occur in the context of hundreds or thousands of competing and cooperative interactions with other lncRNAs in a complex ecosystem that controls signaling in the nucleus. The expected result would include small but cooperative and cumulative effects on a large number of downstream targets, thus displaying itself as a relatively subtle contribution to one or possibly many complex phenotypes.

## Conclusions

Clearly, we are still at the early stages of understanding the full complexity of functional elements encoded in the human genome. However, recent results paint an emerging picture of a very complex regulatory network composed of numerous ncRNAs and their targets. Each ncRNA molecule in this network could potentially regulate hundreds of other RNAs in *trans*. GWAS variants could function by affecting this overarching layer of ncRNA regulation. In fact, the recent examples of this type of network regulation probably represent the tip of the iceberg of its true significance in complex diseases. Therefore, the time is right to bring the two fields together to fully unravel the underlying relationships.

In addition, the theme of ncRNA in disease brings with it an immediate implication for clinical research. If ncRNAs are as intricately involved in underlying disease mechanisms, as the data reviewed here suggest, then clinical transcriptome sequencing (including ncRNAs) has to come to the forefront of biomedical research. Indeed, first indications suggest that ncRNAs represent excellent biomarkers for cancer diagnostics [46,47]. Considering the much larger complexity of ncRNAs compared with coding RNAs, the former represent a gigantic untapped potential for clinically relevant biomarkers, in addition to expanding our basic knowledge of molecular events leading to disease.

## Abbreviations

GWAS: genome-wide association study; lincRNAs: long intergenic non-coding RNAs; lncRNA: long non-coding RNA; ncRNA: non-coding RNA; NHGRI: National Human Genome Research Institute; SNP: single nucleotide polymorphism; vlincRNA: very long intergenic non-coding RNA.

## Competing interests

The authors declare that they have no competing interests.

## Authors’ contributions

GSL and PK jointly wrote the manuscript, and YV performed bioinformatics analysis. All authors read and approved the final manuscript.

## Supplementary Material

Additional file 1 Supplementary Text. Click here for file

Additional file 2 **Supplementary Tables 1-3.** Click here for file
