# Supplementary material for: Dark matter RNA illuminates the puzzle of genome-wide association studies
Source: BMC Med. 2014 Jun 12;12:97. doi: 10.1186/1741-7015-12-97 (PMC4054906; doi:10.1186/1741-7015-12-97)
Supplement: Additional file 1 — Supplementary Text. [file 1741-7015-12-97-S1.pdf]

Supplementary Text

The GWAS database was downloaded from the UCSC Genome Browser database (http://www.genome.ucsc.edu/cgi-bin/hgTrackUi?g=gwasCatalog). Each trait was assigned to a general disease type or a non-disease trait using information about studies provided in the dataset. Sometimes the same GWAS variant was found associated with more than one disease belonging to same disease type, potentially inflating statistics. To avoid such cases, yet to recognize the value of variants found associated with the same disease type in independent studies, we selected entries with the unique combination of the following fields: (1) name of the variant, (2) PubMed record ID and (3) disease type (Supplementary Table 1). Therefore, the same GWAS variant was counted more than once if it was associated with different disease types or with the same disease type but in more than one independent publication. Overlap with genomic annotations was done in a hierarchical fashion: a SNP overlapping multiple genomic annotations was assigned to the annotation with a higher priority. The order of priorities were (from highest to lowest): CDS's, UTR's, promoters of known genes, introns of known genes, lincRNAs (exons and introns), vlincRNAs and remaining Intergenic space, where CDS's (portions of genes that code proteins), UTR's (untranslated gene regions on each side of CDS) and introns were taken from UCSC Genes table (http://www.genome.ucsc.edu/cgi-bin/hgTrackUi?g=knownGene), promoters were treated as genomic regions within ±2000nt from transcription start site of each gene, lincRNAs are taken from lincRNA and TUCP transcripts UCSC table (http://www.genome.ucsc.edu/cgi-bin/hgTrackUi?g=lincRNAsTranscripts), and vlincRNAs are collected from different samples in [1]. For each disease category, the p-value for a skew towards a particular genomic category was calculated as 1-Hyp(k-1,K,n,N), where Hyp is the cumulative Hypergeometric distribution function, k is number of the unique entries (per above) of given disease category in given genomic category, K is the total number of the entries of given disease category, n is the total number of the unique entries in given genomic category, N is 13,559 - the total number of the analyzed entries. The details of analysis are given in the Supplementary Table 2. The p-value is presented as $-10\log_{10}$. The purple bars represent the unique variants (rather than unique entries) (Supplementary Table 3).

1. St Laurent G, Shtokalo D, Dong B, Tackett M, Fan X, Lazorthes S, Nicolas E, Sang N, Triche T, McCaffrey T *et al*: **VlincRNAs controlled by retroviral elements are a hallmark of pluripotency and cancer**. *Genome Biology* 2013, **14**(7):R73.
